# Elimination of Reprogramming Transgenes Facilitates the Differentiation of Induced Pluripotent Stem Cells into Hepatocyte-like Cells and Hepatic Organoids

**DOI:** 10.3390/biology11040493

**Published:** 2022-03-23

**Authors:** Jaemin Jeong, Tae Hun Kim, Myounghoi Kim, Yun Kyung Jung, Kyeong Sik Kim, Sehwan Shim, Hyosun Jang, Won Il Jang, Seung Bum Lee, Dongho Choi

**Affiliations:** 1 Department of Surgery, Hanyang University College of Medicine, Seoul 04763, Korea; jmj1103@gmail.com (J.J.); xo132435@naver.com (T.H.K.); auddghl112@naver.com (M.K.); jyk1986@hotmail.com (Y.K.J.); toopjoo12@gmail.com (K.S.K.); 2 Laboratory of Radiation Exposure & Therapeutics, National Radiation Emergency Medical Center, Korea Institute of Radiological & Medical Science, Seoul 01812, Korea; ssh3002@kirams.re.kr (S.S.); hsjang@kirams.re.kr (H.J.); zzang11@kirams.re.kr (W.I.J.); 3 Hanyang Indang Center of Regenerative Medicine and Stem Cell Research, Hanyang University, Seoul 04763, Korea; 4 Department of HY-KIST Bio-Convergence, Hanyang University, Seoul 04763, Korea

**Keywords:** hiPSCs, transgene-free, Cre-loxP system, hepatocyte differentiation, hepatic organoids

## Abstract

**Simple Summary:**

The elimination of reprogramming transgenes affects the differentiation potential of human induced pluripotent stem cells (hiPSCs) at the early embryonic development stage, but not during the late stage of development into hepatocytes and hepatic organoids. Using an excisable polycistronic lentiviral system (STEMCCA, Cre-loxP system), we generated both transgene-carrying and transgene-free hiPSCs from human fibroblasts and demonstrated that the elimination of transgenes enhances the differentiation potential of iPSCs toward hepatocyte-like cells and the generation of hepatic organoids, exhibiting efficient hepatic differentiation. Our findings thus provide significant insights into the characteristics of iPSC-derived hepatic organoids.

**Abstract:**

Hepatocytes and hepatic organoids (HOs) derived from human induced pluripotent stem cells (hiPSCs) are promising cell-based therapies for liver diseases. The removal of reprogramming transgenes can affect hiPSC differentiation potential into the three germ layers but not into hepatocytes and hepatic organoids in the late developmental stage. Herein, we generated hiPSCs from normal human fibroblasts using an excisable polycistronic lentiviral vector based on the Cre recombinase-mediated removal of the loxP-flanked reprogramming cassette. Comparing the properties of transgene-carrying and transgene-free hiPSCs with the same genetic background, the pluripotent states of all hiPSCs were quite similar, as indicated by the expression of pluripotent markers, embryonic body formation, and tri-lineage differentiation in vitro. However, after in vitro differentiation into hepatocytes, transgene-free hiPSCs were superior to the transgene-residual hiPSCs. Interestingly, the generation and hepatic differentiation of human hepatic organoids (hHOs) were significantly enhanced by transgene elimination from hiPSCs, as observed by the upregulated fetal liver (CK19, SOX9, and ITGA6) and functional hepatocyte (*albumin*, ASGR1, HNF4α, CYP1A2, CYP3A4, and AAT) markers upon culture in differentiation media. Thus, the elimination of reprogramming transgenes facilitates hiPSC differentiation into hepatocyte-like cells and hepatic organoids with properties of liver progenitor cells. Our findings thus provide significant insights into the characteristics of iPSC-derived hepatic organoids.

## 1. Introduction

Hepatocyte transplantation is a widely available approach for treating patients with end-stage liver disease induced by alcohol use, hepatotoxic drugs, or hepatitis viral infections [1,2]. However, the use of human hepatocytes has been hampered by donor organ shortages and difficulty in maintaining and proliferating cells in vitro. As an alternative method to develop cell-based therapies for treating liver disease, much effort has been exerted over the past decade for the potential use of hepatocyte-like cells induced from stem cells. Stem cells, including induced pluripotent stem cells (iPSCs), embryonic stem cells (ESCs), mesenchymal stem cells (MSCs), and chemically derived hepatic progenitors (hCdH), are capable of indefinite self-renewal [2,3,4,5]. More recently, to further mimic the in vivo liver environment, a 3D culture with Matrigel as a cell-extracellular matrix (ECM) was developed for cell differentiation into a hepatic lineage, which forms spherical monolayer epithelium structures called human hepatic organoids (hHOs) that retain the physiological features of the human liver [6]. hHOs have been derived from liver tissue stem cells and pluripotent stem cells, including human iPSCs [6,7,8,9]. Moreover, hHOs retain a fetal-like state capable of high proliferation and bilineage differentiation, such as hepatocytes and cholangiocytes, both in vitro and in vivo [10,11]. This organoid technology with a 3D culture system has emerged as a prospective strategy for developing cell-based therapies for liver diseases.

iPSCs represent a major advance in biomedical research for cell therapy and disease modeling in regenerative medicine owing to their acquisition of self-renewal and pluripotency similar to ESCs [12,13,14,15]. Defined factor-induced reprogramming has mostly been achieved by co-infection with a retroviral vector, which is integrated into the donor genome. However, reactivation of these reprogramming genes upon iPSC differentiation into somatic cells has raised concerns regarding their clinical application because of the induction of oncogenicity and mutagenesis. The production of transgene-free iPSCs is, thus, a critical safety concern for their potential use in regenerative medicine. To make iPSCs more compliant with therapeutic applications without viral vector integration, the original methodology has been advanced through several strategies, including the use of adenoviral vectors [16], non-integrating episomal vectors [17], the elimination of integrated vectors using piggyback transposition [18], and using a single excisable lentiviral vector (STEMCCA, Cre/loxP system) [19,20].

Functionally, in terms of cellular DNA replication, the methods of involving either the integration or non-integration of transgenes do not significantly affect genomic integrity, as indicated by the single-nucleotide polymorphisms (SNPs) and copy number variations (CNVs) between donor cells and iPSCs determined using whole-genome microarrays [21]. However, it has been reported that transgene-free hiPSCs have different transcriptome profiles and epigenetic patterns, which facilitate their differentiation into the three germ layers, as well as into functional cell types such as keratinocytes, blood, and neural cells [22,23,24,25]. Although mRNA-derived iPSCs (a non-integration transgene method) differentiate efficiently into hepatoblasts, which are fetal precursors of hepatocytes, compared with retroviral-derived iPSCs [21], the effect of transgene excision on the potential of hiPSCs to differentiate into hepatocytes, especially into hepatic organoids, remains poorly understood.

In this study, we compared the hepatic differentiation potential and characteristics of hepatic organoids derived from iPSCs after the removal of reprogramming transgenes using the Cre/loxP system.

## 2. Materials and Methods

### 2.1. hiPSC Generation Kit and Antibodies

The human STEMCCA/TAT-Cre bundle used for iPSC generation was purchased from Sigma-Aldrich (SCR545-CRE, Louis, MO, USA). Antibodies against KLF4 (Santa Cruz Biotechnology, sc-20691, Dallas, TX, USA), OCT3/4 (Santa Cruz Biotechnology, sc-5279), SOX2 (Cell Signaling Technology, 4900, Danvers, MA, USA), c-MYC (Santa Cruz Biotechnology, sc-764), NANOG (Cosmo Bio, REC-RCAB0004PF, Tokyo, Janpan), SOX17 (R&D Systems, AF1924), *albumin* (Thermo Fisher Scientific, A80-229A, Waltham, MA, USA), AFP (Abcam, ab3980, Cambridge, UK), HNF4α (Santa Cruz Biotechnology, sc-8987), ASGR1 (Santa Cruz Biotechnology, sc-52623), CYP1A2 (Santa Cruz Biotechnology, sc-53241), and β-actin (Santa Cruz Biotechnology, sc-47778) were used.

### 2.2. Cell Culture

Human fibroblasts (passage 9, GlobalStem Inc. Rockville, MD, USA) were maintained in DMEM supplemented with 10% FBS. hiPSCs were cultured in six-well plates coated with Matrigel in mTeSR1 medium and passaged using ReLeSR; the culture medium was changed daily. mTeSR1 and ReLeSR were provided by STEMCELL Technology, Inc. (Vancouver, BC, Canada).

### 2.3. Generation of Transgene-Free hiPSCs

hiPSCs were generated from fibroblasts according to the manufacturer’s instructions (human STEMCCA/TAT-Cre Bundle kit; Sigma-Aldrich). Briefly, fibroblasts attached overnight were infected using excisable polycistronic lentiviral particles (human LoxP STEMCCA) with 5 µg/mL polybrene for 8 h, and then incubated for 5 days. Infected cells were cultured at 5 × 10^4^ cells/well until the appearance of ESC-like cells; the hiPSC induction media was changed daily (TeSR-E7 media, STEMCELL Technology, Vancouver, BC, Canada). To generate transgene-free hiPSCs, STEMCCA was excised by treatment with TAT-CRE recombinase included in the kit. The resulting STEMCCA excision was confirmed by PCR using genomic DNA extracted from the hiPSCs.

### 2.4. Live-Staining of Cells and Alkaline Phosphatase (ALP) Assay

Live cells were stained using StainAlive TRA1-81 (anti-human, DyLight 488) and SSEA-4 (anti-human, DyLight 550) obtained from STEMGENT Inc. An ALP detection kit (System Biosciences, Palo Alto, CA, USA) was used to determine ALP activity.

### 2.5. Western Blotting

Proteins were extracted using a cell lysis buffer containing 50 mM Tris-HCl (pH 7.4), 150 mM NaCl, 5 mM EDTA, protease inhibitors, and 1% Nonidet P-40. A total of 20 μg of protein were separated by SDS-PAGE, electrotransferred onto membranes, and subsequently probed with the indicated antibodies. An ECL chemiluminescent reagent (GE Healthcare Life Sciences, Piscataway, NJ, USA) was used for detection.

### 2.6. Genomic DNA Purification and Quantitative Real-Time PCR (qPCR)

Genomic DNA from cells was purified using a QIAamp DNA Micro Kit (Qiagen, Valencia, CA, USA). The inserted transgenes were detected by PCR with genomic DNA (50 ng), using specific primers (F: 5′-GGA ACT CTT GTG CGT AAG TCG ATA G-3 and R: 5′-GGA GGC GGC CCA AAG GGA GGA GAT CCG-3). PCR was conducted using 35 cycles (94 °C for 30 s, 60 °C for 30 s, and 72 °C for 40 s). After total RNA was prepared using an RNase Mini Kit (Qiagen), 1 μg of RNA was reverse-transcribed and used for qPCR using the AccuPower RT PreMix kit (Bioneer, Daejon, Korea) and FastStart Essential DNA Green Master (Roche, Indianapolis, IN, USA). GAPDH expression was used to normalize the mRNA expression levels. The primer sequences used are listed in Table 1.

### 2.7. Immunocytochemistry

Cells and organoids fixed with 4% paraformaldehyde were treated with permeabilization buffer containing 1% BSA and 0.1% Triton X-100 in PBS for 1 h at room temperature. After washing thrice with PBS, the sections were incubated with primary antibodies against hNanog (1:200), hSox2 (1:100), hOCT3/4 (1:50), Sox17 (1:100), *albumin* (1:50), AFP (1:100), HNF4α (1:100), ASGR1 (1:50), and CYP1A2 (1:100). Secondary antibodies labelled with either Alexa Fluor 488 (goat anti-rabbit IgG, 1:500; Invitrogen, Waltham, MA, USA) or Alexa Fluor 594 (goat anti-mouse IgG, 1:500, Invitrogen) were used. Images were obtained using a fluorescence microscope (Olympus, Shinjuku, Tokyo, Japan).

### 2.8. Karyotyping of Transgene-Free hiPSCs

For transgene-free hiPSCs, chromosomal analysis was conducted using Giemsa staining (GTG banding). A total of 20 metaphase spreads were evaluated. Euploidy in 20 of 20 cells was defined as normal.

### 2.9. Formation of Embryoid Body (EB) and In Vitro Trilineage Differentiation

The induction of EB formation from either transgene-carrying or transgene-free hiPSCs was investigated by suspending dissociated hiPSC colonies into ultra-low attachment plates. Suspended hiPSCs were incubated for 5 days with EB media comprising hESC media without bFGF (DMEM/F12, 20% knockout serum, 0.1 mM nonessential amino acids, 2 mM GlutaMAX, 50 U/mL penicillin, 50 g/mL streptomycin, 0.1 mM beta-mercaptoethanol). EB images were captured using an inverted light microscope (Olympus). Three germ layer differentiation from both hiPSC lines was induced using an hPSC functional identification kit (R&D Systems, Minneapolis, MN, USA).

### 2.10. Generation and Expansion of hHOs Differentiated from hiPSCs

To induce definitive endoderm (DE) differentiation from hiPSCs, both hiPSCs were sequentially cultured in DE differentiation medium I (1% B27 (Gibco, Waltham, MA, USA), 100 ng/mL Activin A (PeproTech, Rockyhill, NJ, USA), and 50 ng/mL Wnt3A (R&D) in RPMI 1640 medium) for 1 d, followed by DE differentiation medium II (2% B27 and 100 ng/mL Activin A in RPMI 1640 medium). DE cells were further incubated in hepatocyte medium (10 ng/mL FGF-2 (PeproTech) and 20 ng/mL BMP4 (R&D) in HCM medium (Lonza, Walkersville, MD, USA)) for 5 days. For inducing hHOs, single cells (10^4^ cells per well) using TrypLE™ Express Enzyme (Gibco) were embedded into Matrigel as 50 μL droplets and cultured in hHO differentiation medium (1% Glutamax, 1% N2, 1% B27, 10 mM HEPES, 1.25 mM N-acetylcysteine, 10 nM gastrin (Sigma), 10 mM nicotinamide, 5 μM A83–01 (Sigma), 10 μM Forskolin (Sigma), 250 ng/mL R-spondin1 (R&D), 100 ng/mL Wnt3a (R&D), and 50 ng/mL EGF (PeproTech) in Advanced DMEM/F12 medium). Media were changed every 2–3 days and hHOs were passaged at 1–2 weeks at a ratio of 1:5–1:8.

### 2.11. Hepatic Differentiation from hiPSC-Derived HOs

To induce mature hepatocytes from hiPSCs-derived hHOs, hHOs embedded in Matrigel were sequentially cultured in hepatic differentiation medium I (0.1% BSA, 1% Glutamax, 10 mM HEPES, 1% N2, 1% B27, 1.25 mM N-acetylcysteine, 10 mM nicotinamide, 10 nM gastrin, 25 ng/mL BMP7 (R&D), 25 ng/mL FGF4 (R&D), and 50 ng/mL HGF (R&D)) for 4 days, followed by hepatic differentiation medium II (0.1% BSA, 1% B27, 1.25 mM N-acetylcysteine, 0.2 mM L-ascorbic acid trisodium salt (Sigma), 10 μM Y-27632 (Stemcell), 0.5 μM dexamethasone (Sigma), 20 ng/mL HGF, and 20 ng/mL OSM (R&D) in HCM medium) for 3 days. After the induction of the hepatocytes from the hHO, the cells were maintained in HCM as hepatic culture media supplemented with 0.1% BSA, 1% B27, 0.2 mM L-ascorbic acid trisodium salt, 1.25 mM N-Acetylcysteine, 0.5 μM dexamethasone, 1% ITS (Gibco), 100 µM 8-Br-cAMP (Selleck, Houston, TX, USA), 10 μM Y-27632, 10 ng/mL EGF, and 10 µM VK2 (Sigma). The medium was changed every 2–3 days for 8 days.

### 2.12. Statistical Analysis

Quantitative data are shown as mean ± standard deviation (SD) with inferential statistics (*p*-values). Two-tailed *t*-tests were used to evaluate statistical significance (* *p* < 0.05, ** *p* < 0.01, and *** *p* < 0.001).

## 3. Results

### 3.1. Generation and Characterization of hiPSCs from Fibroblasts Using an Excisable Polycistronic Lentiviral System

**Figure 1:**
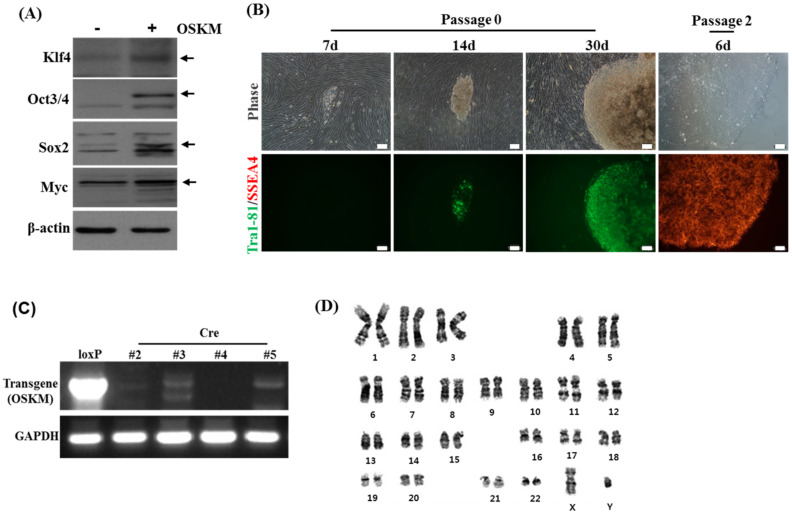
Generation of transgene-eliminated human induced pluripotent stem cells (hiPSCs) derived from fibroblasts. Fibroblasts were transduced with EF1alpha-STEMCCA lenti-4F (*OSKM*). (**A**) Western blotting for *Oct3/4, Sox2, Klf4*, and *c-Myc* in *OSKM* overexpressing fibroblasts. *Beta-actin* was used as the loading control. (**B**) Representative morphology and live-staining of *Tra1-81* antibody (green) during reprogramming, and live-staining of *SSEA1* (red) after passage 2. *Tra-81* and *SSEA4* are known as pluripotent stem cell surface markers. Scale bar = 100 μm. (**C**) PCR analysis of genomic DNA purified from four representative iPS clones among seven clones with the STEMCCA-loxP vector, before and after Cre-mediated removal. (**D**) Karyotyping of hiPSCs generated from fibroblasts.

**Figure 2:**
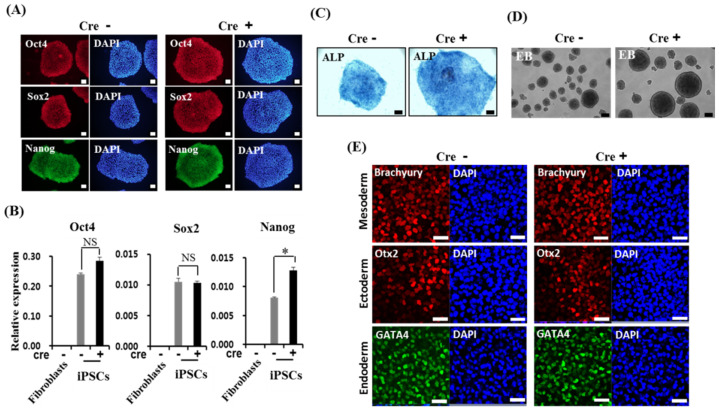
Characterization of transgene-free human induced pluripotent stem cells (hiPSCs) from fibroblasts. (**A**) Immunostaining for pluripotent markers (*Oct4, Sox2,* and *Nanog*) in hiPSCs with (Cre−) or without (Cre+) four factors (*OSKM*) at passage seven. Nuclei were stained with DAPI (blue). Scale bar = 100 μm. (**B**) qPCR analysis of the expression of pluripotent markers such as *Nanog, Oct4,* and *Sox2* in hiPSCs with or without four factors. mRNA expression level of pluripotent markers was calculated relative to that of GAPDH. The data are shown as mean ± SD from triplicate experiments (* *p* < 0.05, two-tailed Student’s *t*-tests). (**C**) Alkaline phosphatase (ALP)-stained colonies of hiPSCs with or without four factors. Scale bar = 100 μm. (**D**) EB formation from hiPSCs with or without four factors for 3 days. Scale bar = 100 μm. (**E**) In vitro differentiation potential from hiPSCs with or without four factors into tri-lineage type cells. Immunostaining of *Brachyury* (red, mesoderm), *Otx2* (red, ectoderm), and *GATA4* (green, endoderm) using antibodies specific for the three germ layers. Nuclei were stained with DAPI (blue). Scale bar = 50 μm. Cre−: hiPSCs with four factors (*OSKM*). Cre+: hiPSCs without four factors (*OSKM*). NS: not statistically significant.

Reprogramming of somatic cells into hiPSCs can be accomplished by the exogenous expression of four factors including *Oct4*, *Sox2*, *Klf4*, and *c-Myc*, referred hereafter as *OSKM* [12,13,14,15]. The elimination of reprogramming transgenes affects the differentiation capacity of hiPSCs at the stage of early embryonic development into three germ layers [23], but not during the late stage of development into hepatocytes and hepatic organoids. Therefore, we first investigated whether the capacity of hiPSCs to differentiate into hepatocytes was modulated by the presence or absence of transgenes in hiPSCs. To generate hiPSCs, human fibroblasts were transfected with human STEMCCA loxP (*OSKM*) lentiviral particles with a loxP site inserted into the 3’ long terminal repeat (LTR) of self-inactivating lentiviral vectors, including the four reprogramming factors. Prior to hiPSC formation, *OSKM* expression was determined using immunoblotting (Figure 1A and File S1). After four weeks of cultivation from transduction and passage, pluripotency was achieved in the cells, as observed by the hESC-like morphology and expression of pluripotent stem cell surface markers such as *SSEA4* and *Tra1-81* (Figure 1B). To excise the integrated transgene of the floxed vector in hiPSCs, several clones were treated with TAT-CRE recombinase protein, resulting in the successful Cre-mediated removal of STEMCCA-loxP in clone #4, as shown by a PCR of genomic DNA (Figure 1C). Further analysis was performed using clone #4, which exhibited a normal karyotype (Figure 1D). After several passages, both clones with or without the transgene (referred to as Cre− and Cre+, respectively) revealed hiPSC characteristics based on the expression of pluripotent markers, including *Oct4, Sox2, Nanog,* and alkaline phosphatase (ALP), as determined by immunostaining, qPCR, and ALP staining (Figure 2A–C). The pluripotency of both clones was confirmed by the EB formation and direct induction of the three germ layers (endoderm, mesoderm, and ectoderm) in vitro (Figure 2D,E). These results indicated that both clones (Cre− and Cre+) acquired the hiPSC phenotype. However, transgene-free hiPSCs revealed a higher expression of Nanog and larger size of EB formation without a meaningful difference in trilineage differentiation potential compared with that of transgene-carrying hiPSCs.

### 3.2. Transgene-Free hiPSCs Showed Enhanced Direct Differentiation to DE and Hepatocyte-like Cells

**Figure 3:**
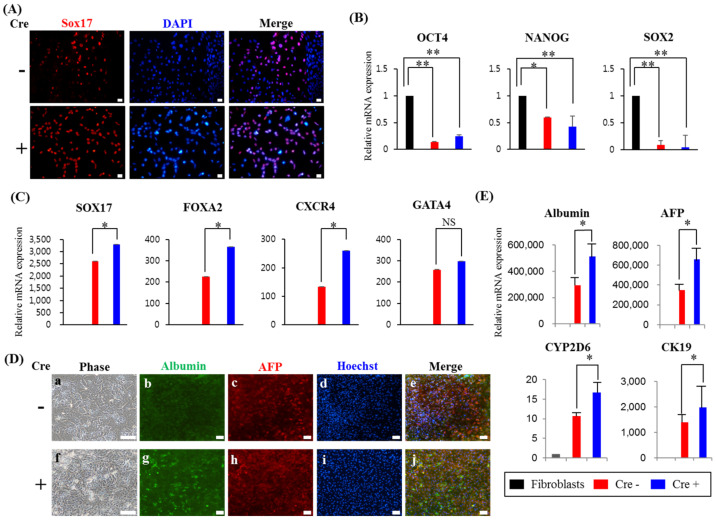
Differentiation of hepatocytes following definitive endoderm differentiation from transgene-free human induced pluripotent stem cells (hiPSCs). Both hiPSCs (Cre−) and hiPSCs (Cre+) were differentiated toward DE, and DE cells were further subjected to hepatocyte differentiation under monolayer culture. (**A**) Representative image of immunostaining for *Sox17,* a definitive endoderm marker, in hiPSCs with (Cre−) or without (Cre+) the four factors. Nuclei were stained with DAPI (blue). Scale bar = 100 μm. (**B**,**C**) qPCR analysis for the expression of pluripotent markers (*Oct4, Sox2* and *Nanog*) and definitive endoderm markers (*SOX17, FOXA2, CXCR4* and *GATA4*) in hiPSCs with or without the four factors. mRNA expression levels of pluripotent or definitive endoderm markers were calculated relative to those in undifferentiated hiPSCs. The data are shown as the mean ± SD from triplicate experiments (* *p* < 0.05, ** *p* < 0.01, two-tailed Student’s *t*-tests). (**D**) Representative image of immunostaining for hepatic markers (*albumin* and AFP) in hiPSCs with or without the four factors. (**E**) Quantitative RT-PCR analysis of hepatic markers (*albumin, AFP, CYP2D6,* and *CK19*) in hiPSCs with or without the four factors. mRNA expression levels of hepatic markers were calculated relative to those in fibroblasts. The data are shown as the mean ± SD from triplicate experiments (* *p* < 0.05, two-tailed Student’s *t*-tests). NS: not statistically significant.

To clarify whether transgenes affect hepatic differentiation, we first attempted to directly differentiate the DE from both iPSCs, as previously reported [26]. We observed that the expression of Sox17, a marker of DE, increased with a reduction in pluripotent stem cell markers during DE differentiation in both clones (Figure 3A,B). Interestingly, hiPSCs (Cre+) showed upregulation of Sox17 and other DE markers, such as *FOXA2* and *CXCR4*, but not *GATA4*, compared to iPSCs (Cre−) (Figure 3A,C). We further extended the differentiation step from DE to hepatocytes (referred to as hepatocyte-like cells). As expected, the expression of hepatocyte-related proteins, such as *albumin* (green) and *AFP* (red), was strongly induced during hepatic differentiation from hiPSCs (Cre+) rather than from hiPSCs (Cre−) (Figure 3D). In support of these findings, hiPSCs (Cre+) also displayed a significant increase in the mRNA expression of *albumin, AFP, CK19,* and *CYP2D6*, which are essential for drug metabolism [27], compared with that in hiPSCs (Cre−) (Figure 3E). These results suggest that the removal of transgenes facilitates the hepatic differentiation of hiPSCs.

### 3.3. Enhanced Generation of hHOs from Transgene-Free hiPSCs

**Figure 4:**
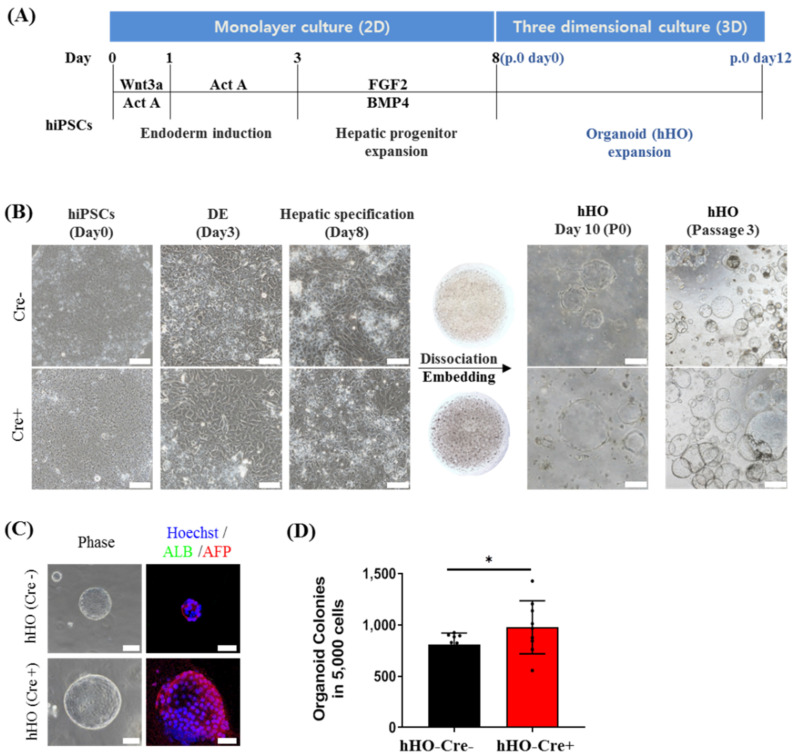
Transgene-free hiPSCs have a higher potential to generate hepatic organoids. Both hiPSCs (Cre−) and hiPSCs (Cre+) were subjected to a differentiation protocol for hepatic organoids (Hos). (**A**) Scheme of hHO generation from hiPSCs. (**B**) Representative image of hiPSC differentiation into the definitive endoderm, hepatic specification with several growth factors, hHOs after 10 days (P0), and subculture (P3). Scale bars = 100 µm (2D), 500 µm (hHOs). (**C**) Representative image of immunostaining for the mature liver marker *albumin* (green) and the fetal liver marker *AFP* (red) in HO generated from either transgene-carrying hiPSCs (Cre−) or transgene-free hiPSCs (Cre+). Hoechst indicates nuclear staining. Scale bar = 100 µm (Phase), 50 µm (Fluorescence). (**D**) Comparison of the number of hHO between Cre− and Cre+ at passage 0. Data are shown as the mean ± SD from triplicate experiments (* *p* < 0.05, two-tailed Student’s *t*-tests).

**Figure 5:**
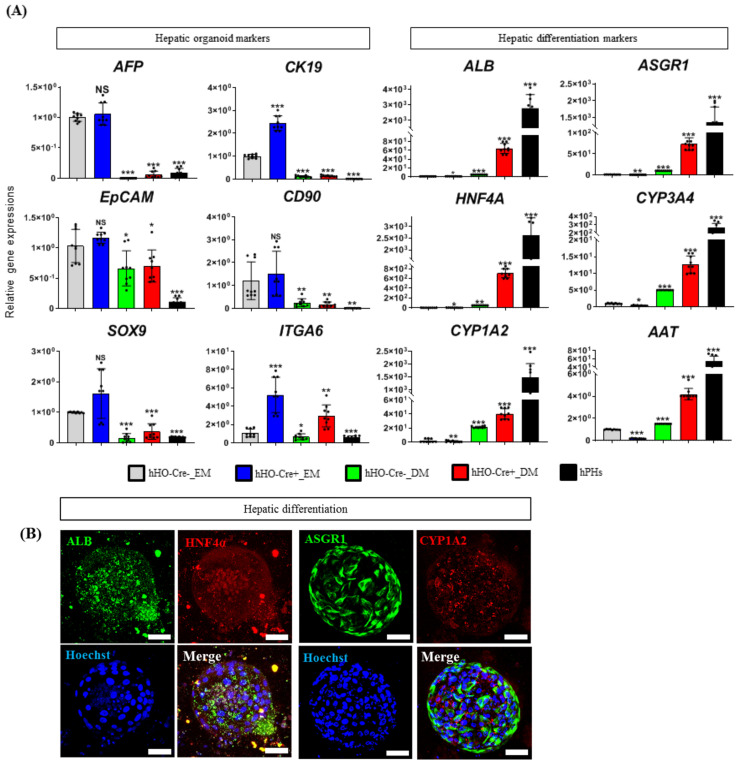
Transgene-free hiPSC-derived hepatic organoids (Hos) (Cre+) have a higher potential of hepatic differentiation. Both hiPSCs (Cre−) and hiPSCs (Cre+) were subjected to hepatic differentiation. (**A**) qPCR analysis of the expression of fetal liver markers (*AFP, CK19, EpCAM, CD90, SOX9,* and *ITGA6*) and mature liver markers (*albumin, ASGR1, HNF4α, CYP3A4, CYP1A2,* and *AAT*) in hHO (Cre−) and hHO (Cre+) following incubation in expansion media and hepatic differentiation media DM, respectively. mRNA expression levels were calculated relative to *GAPDH*. The data are shown as the mean ± SD from triplicate experiments (* *p* < 0.05, ** *p* < 0.01, and *** *p* < 0.001, two-tailed Student’s *t*-tests). (**B**) Representative image of immunostaining for mature liver markers, *albumin* (green), *HNF4α* (red), *ASGR1* (green), and *CYP1A2* (red) in hHO (Cre+)-derived hepatocytes. Hoechst indicates nuclear staining. Scale bar = 500 nm. NS: not statistically significant.

Recently, liver organoid technology has emerged as a powerful and revolutionary strategy enabling the study of human development and disease, owing to the retention of fetal-like states including proliferative and differentiative abilities in prolonged culture [6,10,11]. In particular, hHOs are derived from pluripotent stem cells, including hiPSCs [7,8,9]. As transgene removal facilitated the hepatic differentiation of hiPSCs, we investigated its effect on the induction of hHOs from hiPSCs. The protocol for hiPSC-derived hepatic organoids was previously described by Wang et al. [11] with some modifications. Briefly, after DE induction from hiPSCs using activin A and *Wnt3A*, the cells were further incubated with *BMP4* and *FGF2* for an additional 5 days for the hepatic lineage differentiation in monolayer (2D) culture. After the dissociation of the hepatic progenitor cells, they were treated with HO induction media for 10 days in a 3D culture system with Matrigel to generate hHOs, as shown in Figure 4A,B. The resulting organoids appeared as hollow spherical structures, as seen in Figure 4C. Furthermore, a subculture of hHOs revealed the expression of *AFP*, a fetal liver marker, but not *albumin*, which is a marker of the mature liver (Figure 4C). Interestingly, the size and generation efficiency of hHOs derived from hiPSCs (Cre+) were higher than those of hHOs derived from hiPSCs (Cre−) (Figure 4C,D) upon 3D culture for 12 days after embedding 5000 cells in Matrigel. Real-time PCR analysis further revealed that hHOs (Cre+) significantly upregulated the expression of other fetal liver markers, including *CK19, SOX9,* and *ITGA6*, compared with those in hHOs (Cre−) upon culture with expansion media (Figure 5A). Furthermore, upon hepatic differentiation, the expression of fetal liver markers was dramatically decreased in both hHOs; however, hHOs (Cre+) revealed a considerably stronger increase in *albumin, ASGR1, HNF4α, CYP3A4, CYP1A2,* and *AAT* (all known markers of functional hepatocytes) than that in hHOs (Cre−) (Figure 5A) as indicated by immunofluorescence staining (Figure 5B). Overall, hiPSCs (Cre+) displayed a higher generation and hepatocyte-forming potential than hiPSCs (Cre−), indicating that the elimination of reprogramming transgenes facilitates the differentiation of hiPSCs into hepatic organoids.

## 4. Discussion

iPSC-derived hepatocytes have emerged as an attractive approach for cell therapy and disease modeling for drug screening in liver diseases [2,28]. Recently, as a new differentiation method using 3D culture, hHOs derived from hiPSCs were found to recapitulate enhanced hepatocyte metabolic function and to be more closely related to the physiology of hepatocytes in the liver [7,8,9]. In this study, we expanded on these findings to demonstrate that the generation of hepatocyte-like cells and hepatic organoids with the properties of liver progenitor cells can be facilitated by the removal of reprogramming transgenes in hiPSCs. This was achieved by using an excisable polycistronic lentiviral system (STEMCCA, Cre-loxP system), a well-defined experimental model [23,24,25].

We established transgene-carrying (Cre−) and transgene-free (Cre+) iPSC lines following the Cre recombinase-mediated removal of the loxP-flanked reprogramming cassette, as demonstrated by genomic DNA PCR. We confirmed that both clones (Cre− and Cre+) obtained the characteristics of pluripotent stem cells through the analysis of *SSEA4*, *Tra1-81, Oct4, Sox2*, and *Nanog* expression, which are typical pluripotent markers; they also showed pluripotency, which is the ability to differentiate into all three germ layers, including the endoderm, mesoderm, and ectoderm [12,29]. Interestingly, our comparative study revealed that transgene-free hiPSCs expressed higher levels of *Nanog* and showed larger EB formation than transgene-carrying hiPSCs. Considering that EB size modulates the differentiation processes of stem cells for the effective and rapid generation of differentiated cells from pluripotent stem cells [30,31], transgene-free hiPSCs are useful in the context of hiPSC-derived cell transplantation.

Several studies have demonstrated the differentiation of hepatocytes from hiPSCs using a non-integration system [32,33,34]. However, a systemic comparison of the differentiation potential of hiPSCs into hepatocytes with or without reprogramming transgenes has not been reported, even though the three germ layers (early stage of embryonic development) are efficiently differentiated after the removal of reprogramming transgenes from iPSCs [23]; further, some controversial reports indicate that the removal of transgenes from iPSCs results in efficient differentiation into the ectoderm [23,35] but not into the neural lineage [36]. On the other hand, Steichen et al. used different reprogramming strategies, such as retrovirus (integration) and messenger RNA (non-integration), to generate iPSCs and reported that non-integrated iPSCs differentiated effectively into hepatoblasts (fetal precursors of hepatocytes) [21]. Consistently, hepatocyte differentiation was also enhanced from non-integrated iPSCs generated using an excisable polycistronic lentiviral system. The advantage of our reprogramming method is that it allows comparison of the properties of transgene-carrying and transgene-free hiPSCs with the same genetic background. Therefore, it is worth noting that our findings demonstrated an enhanced differentiation potential of transgene-free hiPSCs (Cre+) into hepatocytes following enhanced DE differentiation in monolayer culture (2D).

To improve the functionality of hepatocyte-like cells differentiated from pluripotent stem cells using the approach of 3D culture with Matrigel, much effort has been devoted to generate hHOs, which represent a spherical monolayer epithelium. To induce hHOs from iPSCs, we adopted the strategy described by Wang et al. [11]. The HOs derived from hESCs in this study were shown to be expandable with the maintenance of the fetal-liver state for potential clinical applications by overcoming previous restrictions, such as limited expansion in vitro and less maturation of hepatocytes from hHOs generated in different ways upon hepatic differentiation [6,37]. Indeed, our hHOs derived from both hiPSCs (Cre− and Cre+) exhibited the hHO phenotype, including spherical morphology and the expression of fetal liver marker (AFP), but not mature liver marker (albumin), along with the ability to differentiate into mature hepatocytes. Interestingly, the elimination of transgenes from hiPSCs also increased the size and number of hHOs, which is beneficial for the scalable generation of hHOs. Another remarkable finding was that hepatic maturation of hHOs could be improved by eliminating reprogramming transgenes in hiPSCs. The reason for this is not clear, but may be partly explained by the reactivation of residual transgenes in hiPSCs upon the induction of either hepatocytes or HOs. Furthermore, the hHOs derived from transgene-free iPSCs (Cre+) in our study also revealed long-term expansion and effective hepatic maturation compared with those of hepatocyte-like cells differentiated from hiPSCs (Cre+) under 2D culture [38]. Therefore, transgene-free iPSC-derived hHOs, which display successful expansion, may contribute to the mass production of hepatocytes required for drug discovery, disease modelling, and cell therapy applications.

## 5. Conclusions

Comparative analysis of the characteristics of transgene-carrying and transgene-free hiPSCs revealed that the elimination of transgenes is favorable for hepatic differentiation and liver organoid maturation. Moreover, the enhanced generation and size of liver organoids were accomplished using transgene-free hiPSCs. This finding provides a new strategy for the efficient production of functional hepatic cells and could be useful for basic research as well as for cell therapies.

## Figures and Tables

**Table 1:** The lists of primer sequence for qPCR analysis.

Gene	Forward (5′–3′)	Reverse (5′–3′)
*OCT4*	GATGTGGTCCGAGTGTGGTT	AGCCTGGGGTACCAAAATGG
*SOX2*	GCCCTGCAGTACAACTCCAT	GACTTGACCACCGAACCCAT
*NANOG*	TGAACCTCAGCTACAAACAG	TGGTGGTAGGAAGAGTAAAG
*SOX17*	ACTGCAACTATCCTGACGTG	AGGAAATGGAGGAAGCTGTT
*FOXA2*	GCAGATACCTCCTACTACCA	GAAGCAGGAGTCTACACAGT
*CXCR4*	CTTCTACCCCAATGACTTGTGG	AATGTAGTAAGGCAGCCAACAG
*GATA4*	CTACAGGGGCACTTAACCCA	AGAGCTGAATCGCTCAGAGC
*ALBUMIN*	CACAGAATCCTTGGRGAACAGG	ATGGAAGGTGAATGTTTCAGCA
*AFP*	AGACTGCTGCAGCCAAAGTGA	GTGGGATCGATGCTGGAGTG
*CK19*	TCCGAACCAAGTTTGAGACG	CCCTCAGCGTACTGATTTCC
*EPCAM*	GAACAATGATGGGCTTTATG	TGAGAATTCAGGTGCTTTTT
*CD90*	CTAGTGGACCAGAGCCTTCG	ACAGGGACATGAAATCCGTG
*SOX9*	GAGGAAGTCGGTGAAGAACG	ATCGAAGGTCTCGATGTTGG
*ITGA6*	TCGCTGGGATCTTGATGCTTGC	TGAGCATGGATCTCAGCCTTGTGA
*ASGR1*	CAGCAACTTCACAGCCAGCA	AGCTGGGACTCTAGCGACTT
*HNF4A*	CCAAAACCCTCGTCGACATG	GCACATTCTCAAATTCCAGG
*CYP1A2*	CGGACAGCACTTCCCTGAGA	AGGCAGGTAGCGAAGGATGG
*CYP3A4*	TTCAGCAAGAAGAACAAGGACAA	GGTTGAAGAAGTCCTCCTAAGC
*AAT*	TATGATGAAGCGTTTAGGC	CAGTAATGGACAGTTTGGGT
*GAPDH*	GGACTCATGACCACAGTCCATGCC	TCAGGGATGACCTTGCCCACAG

## Data Availability

Data are available within the article.

## Supplementary Materials

The following are available online at https://www.mdpi.com/article/10.3390/biology11040493/s1, File S1: Original western blot images.
