# Supplementary figures and images for: Elimination of Reprogramming Transgenes Facilitates the Differentiation of Induced Pluripotent Stem Cells into Hepatocyte-like Cells and Hepatic Organoids
Source: Biology (Basel). 2022 Mar 23;11(4):493. doi: 10.3390/biology11040493 (PMC9030920; doi:10.3390/biology11040493)

Figure S1

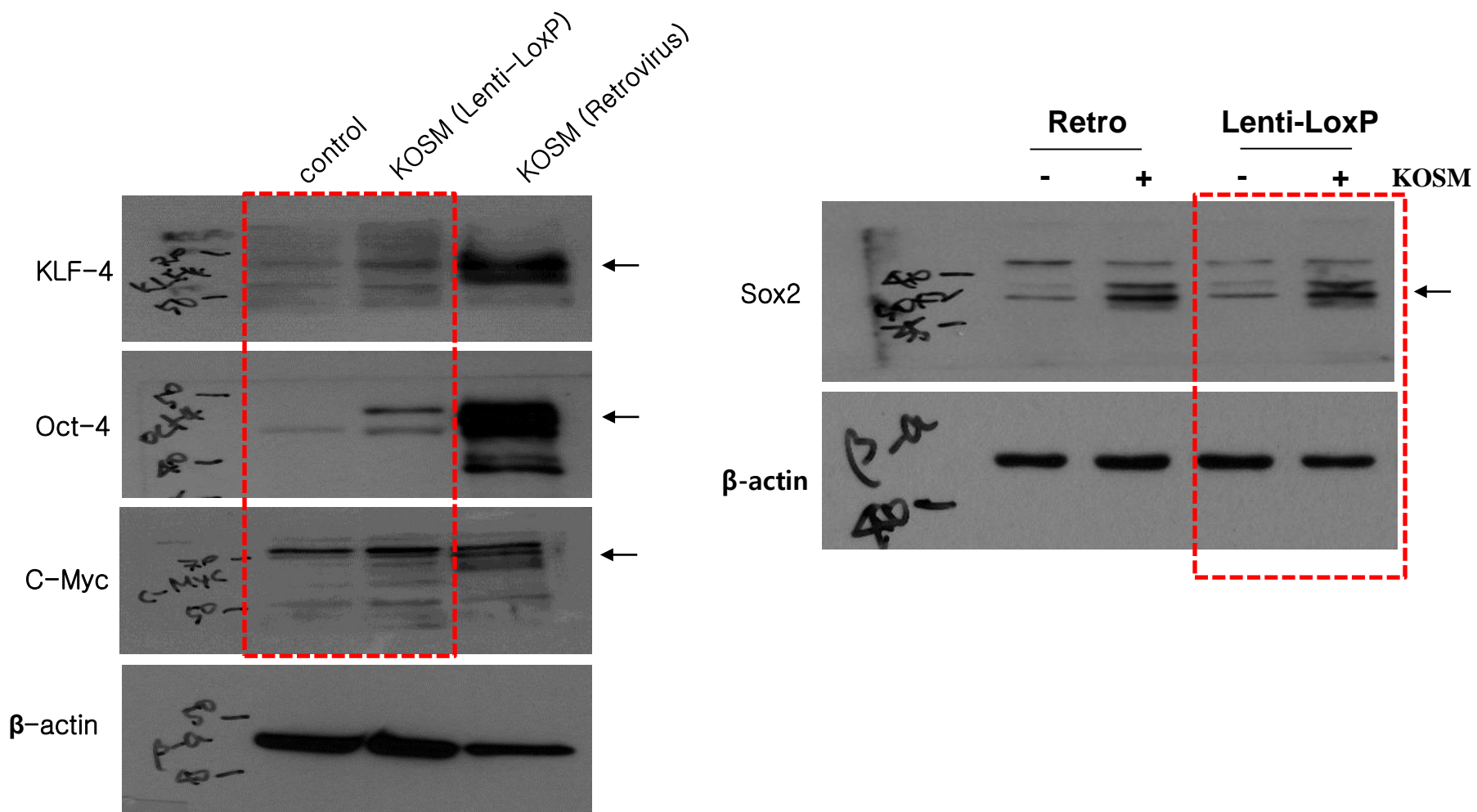

**Figure S2**

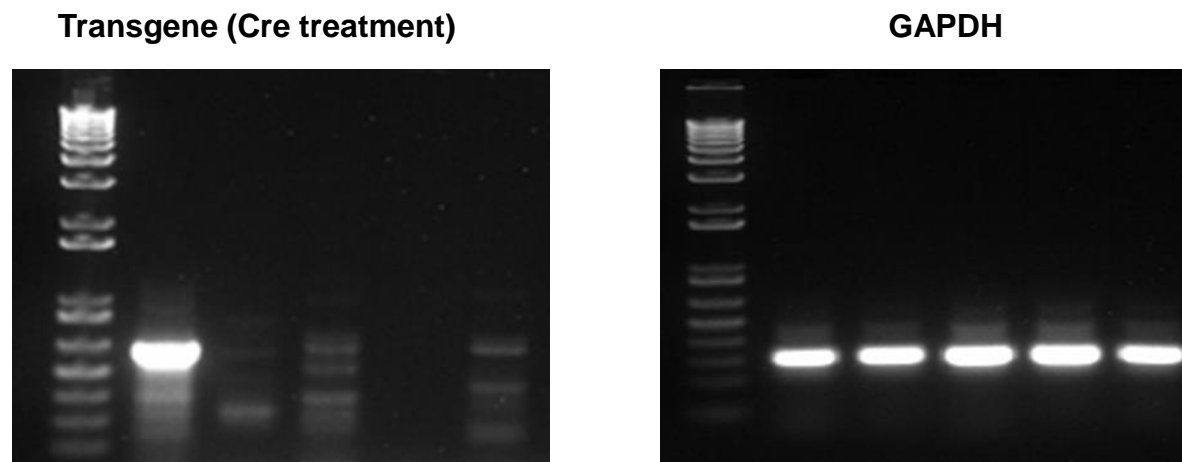

Supplement: Supplementary file 1 [file biology-11-00493-s001.zip › biology-1573801-supplementary materials.pdf]
